# Dynamics of Intracellular Polymers in Enhanced Biological Phosphorus Removal Processes under Different Organic Carbon Concentrations

**DOI:** 10.1155/2013/761082

**Published:** 2013-12-04

**Authors:** Lizhen Xing, Li Ren, Bo Tang, Guangxue Wu, Yuntao Guan

**Affiliations:** ^1^School of Municipal and Environmental Engineering, Shandong Jianzhu University, Jinan, Shandong, 250101, China; ^2^Key Laboratory of Microorganism Application and Risk Control (MARC) of Shenzhen, Graduate School at Shenzhen, Tsinghua University, Shenzhen, Guangdong, 518055, China

## Abstract

Enhanced biological phosphorus removal (EBPR) may deteriorate or fail during low organic carbon loading periods. Polyphosphate accumulating organisms (PAOs) in EBPR were acclimated under both high and low organic carbon conditions, and then dynamics of polymers in typical cycles, anaerobic conditions with excess organic carbons, and endogenous respiration conditions were examined. After long-term acclimation, it was found that organic loading rates did not affect the yield of PAOs and the applied low organic carbon concentrations were advantageous for the enrichment of PAOs. A low influent organic carbon concentration induced a high production of extracellular carbohydrate. During both anaerobic and aerobic endogenous respirations, when glycogen decreased to around 80 ± 10 mg C per gram of volatile suspended solids, PAOs began to utilize polyphosphate significantly. Regressed by the first-order reaction model, glycogen possessed the highest degradation rate and then was followed by polyphosphate, while biomass decay had the lowest degradation rate.

## 1. Introduction

Eutrophication means the overgrowth of algae and cyanobacteria, and, after their death, it causes water pollution by the depletion of oxygen and the release of toxins. Phosphorus in discharged wastewater is one of the main elements contributing to eutrophication and the worsening water quality. In order to reduce or control eutrophication of water bodies, enhanced biological phosphorus removal (EBPR) has been applied widely for phosphorus removal from wastewaters [1]. In EBPR, alternative anaerobic and aerobic phases are adopted and polyphosphate accumulating organisms (PAOs) with excess phosphorus accumulation ability will be enriched [1]. During the anaerobic phase, PAOs take up organic carbons such as acetate and propionate and store them as intracellular polymers such as poly-*β*-hydroxybutyrate (PHB), with polyphosphate as the energy source and glycogen as the reducing power source [1]. In the liquid, the concentration of organic carbon decreases while the concentration of phosphate increases. During the aerobic phase, PAOs synthesize new organisms, restore polyphosphate, and replenish glycogen with stored PHB as the energy and organic carbon sources [1]. In the liquid, phosphorus concentration decreases. PAOs can accumulate polyphosphate with proportions to the total dry biomass weight in the range of 4–15%, which is much higher than that of 2% of general microorganisms [2]. Phosphorus will be removed from wastewater by removing residue activated sludge with high phosphorus content from the wastewater treatment system.

Polyphosphate, PHB, and glycogen are three important polymers of PAOs in EBPR, and the accurate analysis of these components plays an important role in elucidating their dynamics in EBPR. For polyphosphate and PHB, there are some well-recognized analytical methods, while for glycogen, usually, the carbohydrate in activated sludge is converted to glucose under acidic conditions by heating and then analyzed by high-pressure liquid chromatography (HPLC) or spectrophotometer [3]. By this method, the concentration of glycogen inside the biomass will be overestimated by around 40% [3], because carbohydrate in the biomass includes not only intracellular glycogen but also extracellular carbohydrate in extracellular polymeric substances (EPS). Therefore, so as to accurately describe dynamics of intracellular polymers of PAOs, intracellular and extracellular polymers should be differentiated.

During weekend and wet seasons, EBPR may experience deterioration or even failure due to the overflow or the low organic carbon concentration, and this phenomenon is named as the “monday peak” [4, 5]. Ahn et al. [6] found that, after being shocked from the low organic carbon, 20 days were required for PAOs to recover to the normal condition. How to ensure the stable operation of EBPR during low organic carbon conditions is one of the important tasks for wastewater treatment. Performance of EBPR depends on not only the amount of PAOs acclimated but also on the polymers of PAOs due to their important function during biochemical metabolism of PAOs. Most of previous studies have focused on the dynamics of PHB [7] while less focused on glycogen or polyphosphate. As mentioned above, glycogen and polyphosphate also play very important roles in EBPR. Therefore, for further studies, it is necessary to examine dynamics of all these polymers of PAOs in EBPR studies. Under adequate organic carbon conditions, microorganisms will experience excess biomass production and endogenous respiration is not obvious. However, under endogenous conditions, biomass production will be affected and endogenous respiration of PAOs may be dominated. For polymer dynamics under endogenous respiration conditions, there are some contrary conclusions. Lopez et al. [8] obtained that PAOs degraded polyphosphate during the initial several days, while Yilmaz et al. [9] obtained that polyphosphate was released within one day. Therefore, examining dynamics of polymers of PAOs is an important aspect to maintain activities of PAOs [10, 11], and this should be further investigated.

In this study, metabolism of PAOs and dynamics of polymers under different organic carbon concentrations were examined so as to elucidate the function of polymers in EBPR. In addition, dynamics of polymers under endogenous respiration conditions was also investigated to provide some clues for controlling and adjusting the EBPR during low organic carbon shocking conditions.

## 2. Materials and Methods

### 2.1. PAOs Acclimation

PAOs were acclimated in two sequencing batch reactors (SBRs) with different influent organic carbon concentrations at 25°C. One SBR (SBR-L) was supplied with a low sodium acetate (NaAc) concentration (chemical oxygen concentration, named as COD, of around 200 mg/L) and the other SBR (SBR-H) with a high NaAc concentration (COD of around 400 mg/L). The effective SBR working volume was 6 L and the SBR phases of fill, mixing, aeration, settlement, and withdrawal were controlled by timers. The SBRs were operated 4 cycles per day and each cycle included 120 min of anaerobic phase with 10 min of fill phase, 180 min of aerobic phase, 40 min of settlement phase, and idle/withdrawal of 20 min. During the fill phase, influent wastewater of 3 L was pumped by peristaltic pumps into the reactor. Each day, at the same time before the settlement phase, 600 mL of mixed liquor was withdrawn from the reactor to maintain the sludge retention time of around 10 days. The SBRs were seeded with activated sludge taken from Nanshan wastewater treatment plants in Shenzhen, China.

Synthetic wastewater was treated in both reactors. The synthetic wastewater for SBR-L was comprised of NaAc of 255 mg/L, Na_2_HPO_4_ of 91.6 mg/L, NH_4_Cl of 76.5 mg/L, CaCl_2_·2H_2_O of 14 mg/L, MgSO_4_·5H_2_O of 90 mg/L, trace elements of 0.12 mL/L, and yeast extract of 10 mg/L. For the SBR-H, only the NaAc concentration was changed to 510 mg/L, while other components were the same as those of the SBR-L. The components of trace elements were made according to those of Smolders et al. [12].

### 2.2. Batch Experiments

**Figure 1:**
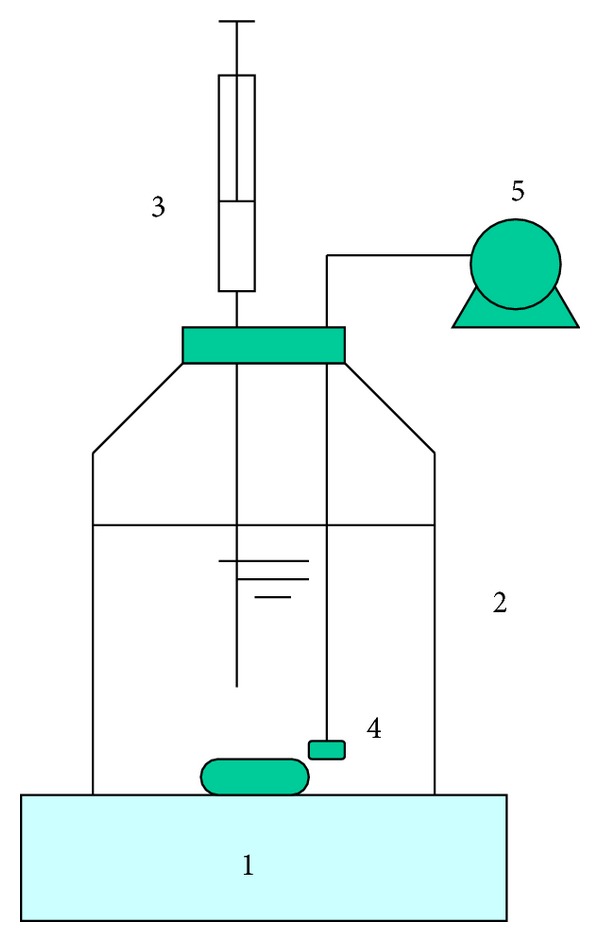
Diagram of the batch experiment reactor. (1) Magnetic stirrer; (2) reactor; (3) liquid sampler; (4) aeration stone; (5) air pump. 4 and 5 only worked during the aerobic condition.

Batch experiments were carried out at 25°C with activated sludge taken from the parent SBRs at steady state. Each batch experiment included activated sludge taken from SBR-H and SBR-L, respectively. For each batch experiment, 400 mL of mixed liquor was withdrawn from the parent SBRs and then filled in a 600 mL capped glass flask (Figure 1). On the cap, several ports were made for sampling, aeration, and so forth. The ports were sealed with tubes for taking samples and so forth. Each batch experiment was replicated and only average results were presented.

In order to examine dynamics of polymers of PAOs under anaerobic conditions, excess organic carbon was supplied and dynamics of different parameters were examined including glycogen, PHB, acetate, and orthophosphate (PO_4_^3−^-P). At the beginning of the experiment, acetate was supplied to the two batch reactors with the initial concentration of 1000 mg/L and then capped to start the experiment. Samples were taken at intervals of 10 min or 15 min.

Endogenous experiments were carried out under both anaerobic and aerobic conditions to examine dynamics of polymers of PAOs. For the anaerobic endogenous experiment, activated sludge mixed liquor taken from the parent reactor was placed in glass flasks directly with an initial nitrogen gas purging for 5 minutes. For the aerobic endogenous experiments, activated sludge mixed liquor taken from the parent SBRs was aerated constantly. Samples were taken at intervals of 12 or 24 hours and each endogenous experiment lasted for 168 hours.

### 2.3. Analytical Methods

Volatile suspended solids (VSS), suspended solids (SS), ammonium nitrogen (NH_4_^+^-N), and PO_4_^3−^-P were measured according to standard methods for the examination of water and wastewater [13].

PHB was measured with the methods of Karr et al. [14] and Rodgers and Wu [15]. Total carbohydrate of biomass was measured according to Lanham et al. [16]: (a) 2 mL of mixed liquor sample was added into a glass tube with 1 mL of deionized water and 0.3 mL of 6 M HCl and then mixed; (b) the mixer was digested at 100°C for 2 h; (c) after cooling down to room temperature, the digested liquor was centrifuged at 12000 rpm for 2 min, and the supernatant was taken for the measurement of glucose by HPLC. Extracellular carbohydrate was extracted according to the method of Li and Yang [17] and Sponza [18]: (a) 2 mL of mixed liquor was heated at 60°C for 30 min; (b) the heated samples were then centrifuged at 12000 rpm for 2 min, and EPS was released from the biomass to the supernatant. The extracellular carbohydrate inside the supernatant was then pretreated as that of the total carbohydrate and then analyzed by HPLC.

PHB, NaAc, and glucose were measured with the HPLC equipment (Shimadzu LC-20A, Japan). PHB and acetate were measured with the UV detector at 210 nm, while glucose was measured with the RID 10-A detector. All these parameters were measured using the Aminex column (HPX-87H, Bio-Rad, USA). The testing conditions used during the HPLC testing were (a) the mobile phase of 0.1% sulfuric acid at the flow rate of 0.6 mL/min; (b) the column temperature of 40°C; (c) the detector cell temperature of 40°C; (d) the injection volume of 20 *μ*L for PHB and the testing duration of 35 min; while those for glucose of 50 *μ*L and 15 min and for acetate of 20 *μ*L and 20 min, respectively.

## 3. Results and Discussion

### 3.1. Acclimation of PAOs and Dynamics of Polymers in Typical Cycles

By using two different influent acetate concentrations, one with a high influent acetate concentration of SBR-H and the other of SBR-L, after 60 days of acclimation, the two SBRs reached steady state. Under steady state, for SBR-H, the SS was 2345 ± 60 mg/L, VSS was 1725 ± 68 mg/L, and the effluent PO_4_^3−^-P was 5.42 ± 0.4 mg/L, while for SBR-L, the SS was 1320 ± 50 mg/L, VSS was 930 ± 56 mg/L, and the effluent PO_4_^3−^-P was 8.28 ± 0.5 mg/L. In spite of the relatively high effluent PO_4_^3−^-P concentrations in both reactors, which could be due to the high influent PO_4_^3−^-P concentration applied, a high phosphorus content in the biomass was obtained for both reactors. The phosphorus content inside the dry biomass was 9.2% in SBR-H and 13.2% in SBR-L. The sludge yield coefficient was 23.6 mg SS/g COD or 17.5 mg VSS/g COD in SBR-H and was 26.5 mg SS/g COD or 18.6 mg VSS/g COD in SBR-L.

**Figure 2:**
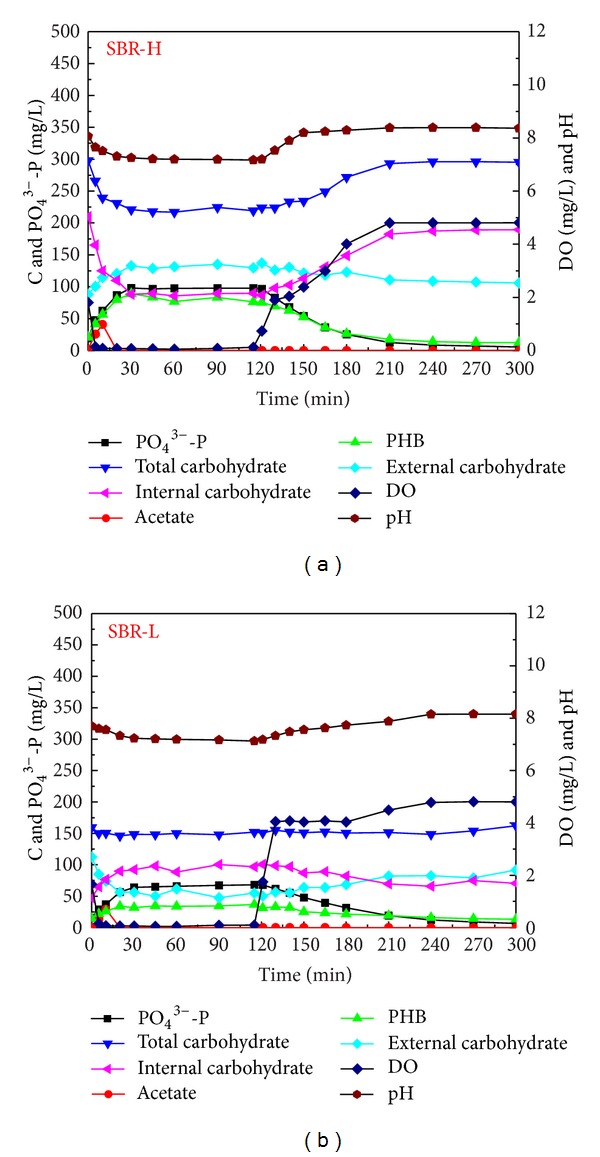
Dynamics of different parameters in typical cycles of SBR-H and SBR-L.

Dynamics of parameters in typical cycles in both reactors are shown in Figure 2. Typical EBPR characteristics were observed in both reactors. In SBR-H, during the anaerobic phase, PHB reached the peak in the initial 30 min with the value of 54.1 mg PHB-C/g VSS; the released PO_4_^3−^-P was 59.6 mg P/g VSS and the concentration of glycogen decreased to 53.5 mg C/g VSS. In SBR-L, in the anaerobic phase, PHB reached the peak value of 34.1 mg PHB-C/g VSS at minute 45; the released PO_4_^3−^-P was 64.26 mg P/g VSS and the concentration of glycogen decreased to 49.8 mg C/g VSS.

In spite of different influent acetate concentrations applied in both reactors, similar sludge yield coefficients were obtained in SBR-H and SBR-L, indicating that sludge production was not significantly affected by the influent organic carbon concentrations. However, a slightly higher phosphorus content was obtained of 13.2% in SBR-L than that of 9.2% in SBR-H. In addition, anaerobic phosphorus release potential was similar in both reactors, with values of 54.6 mg P/g VSS in SBR-H and of 58.9 mg P/g VSS in SBR-L. These results showed that, in the anaerobic and aerobic alternating system, a low influent organic carbon concentration favoured the acclimation of PAOs, which could be due to the high competition ability of PAOs compared with their competitors of glycogen accumulating organisms under low organic carbon conditions. Similar results were also obtained by Tu and Schuler [19].

### 3.2. Anaerobic Dynamics of Polymers of PAOs with Excess Supply of Organic Carbons

**Figure 3:**
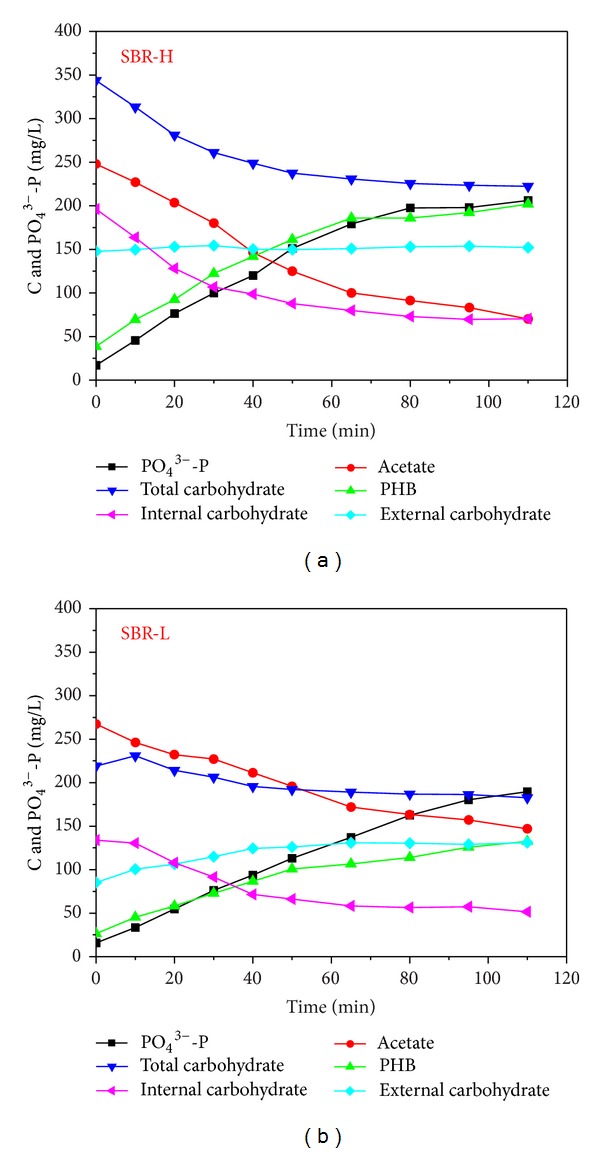
Anaerobic dynamics of polymers of PAOs with excess supply of organic carbons.

Anaerobic dynamics of polymers of PAOs with excess supply of organic carbons is shown in Figure 3. From Figure 3, it is shown that, from minute 50, a high variation in the concentration of various parameters occurred and the regressed biokinetics of polymers is given in Table 1 with durations from minutes 0 to 50 (Phase A) and from minutes 50 to 115 (Phase B).

After supply with excess acetate, PAOs degraded glycogen for supplying reducing power and partial energy. In SBR-H, the total carbohydrate decreased from 189.8 mg C/g VSS to 122.8 mg C/g VSS and the intracellular carbohydrate decreased from 108.3 mg C/g VSS at minute 0 to 38.9 mg C/g VSS at minute 50, while the extracellular carbohydrate kept relatively stable at 82.8 ± 2.2 mg C/g VSS; the decreased intracellular carbohydrate concentration was 69.5 mg C/g VSS during the whole reaction phase. In SBR-L, the total carbohydrate decreased from 238.0 mg C/g VSS to 198.5 mg C/g VSS and the intracellular carbohydrate decreased from 145.5 mg C/g VSS to 56.0 mg C/g VSS, while the extracellular carbohydrate slightly increased with the average concentration of 128.1 ± 29.8 mg C/g VSS; the decreased intracellular carbohydrate concentration was 89.7 mg C/g VSS during the whole reaction phase. These results showed that the intracellular carbohydrate was mainly used for biochemical metabolism during the anaerobic phase, consistent with previous studies, such as that of Wu and Rodgers [20]. This also shows that it is necessary to differentiate the intracellular and extracellular carbohydrate for investigating dynamics of polymer in EBPR. For the extracellular carbohydrate, a slightly higher concentration of 128.3 mg C/g VSS existed in SBR-L than that of 82.8 mg C/g VSS in SBR-H. Extracellular carbohydrate is one main component of EPS [17, 18], and the above results showed that a low influent organic carbon condition induced a high extracellular carbohydrate production, which might be due to the fact that EPS production was a response to low nutrient condition as a protective mechanism for experiencing unfavourable low organic carbon conditions.

By comparing the regressed data in Table 1, during Phase A, *r*_PHB-C/NaAc-C_ was similar in both reactors, indicating that the acetate could be taken up and stored as PHB efficiently, while, during Phase B, a slightly high *r*_PHB-C/NaAc-C_ value was obtained in SBR-H. In spite of a slight variation of *r*_Glycogen-C/NaAc-C_, there was no significant difference in both reactors during both phases. During the whole reaction phase, *r*_PO4-P/NaAc-C_ in SBR-L was higher than that in SBR-H, indicating a high energy requirement from polyphosphate degradation in SBR-L, which showed that there might be high PAO activities or a higher number of PAOs in SBR-L than that in SBR-H. The calculated mole ratios between the released phosphorus and the utilized acetate were 0.40 mol P/mol C in SBR-L and 0.28 mol P/mol C in SBR-H during Phase A and were 0.14 mol P/mol C and 0.16 mol P/mol C during Phase B. During Phase A, a high reaction rate existed due to the existence of high external organic carbons, and the calculated polyphosphate requirement was in the theoretical range of 0.25–0.75 mol P/mol C [12], while during Phase B, due to the limitation of intracellular carbohydrate and the slow activity of PAOs, the biokinetics were relatively slow and were different from those of Phase A.

### 3.3. Dynamics of Polymers of PAOs during Anaerobic and Aerobic Endogenous Respirations

**Figure 4:**
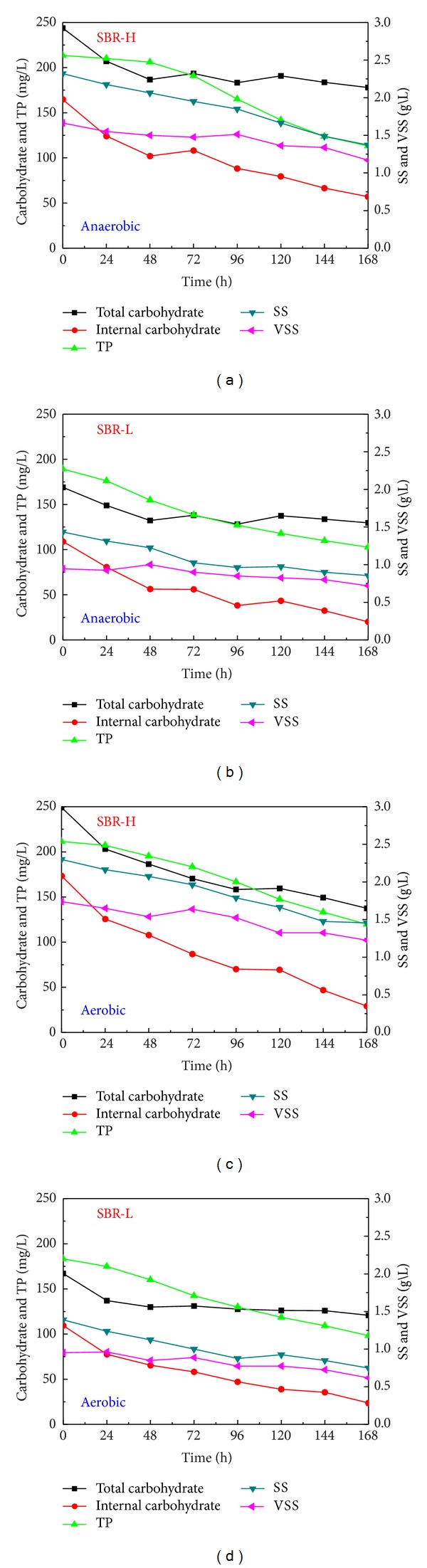
Dynamics of polymers of PAOs and sludge concentrations during anaerobic and aerobic endogenous respirations.

Under anaerobic and aerobic endogenous respiration conditions, dynamics of polymers of PAOs and sludge concentrations are shown in Figure 4.

Under starve conditions, microorganisms will experience endogenous respiration [8, 21], and except the reduction in the biomass concentration, consumption of polymers (for PAOs, glycogen, PHB, etc.) also occurs to provide the required maintenance energy. In SBR-H, during the anaerobic endogenous respiration, different utilization modes of polyphosphate and glycogen occurred, with the main utilization of glycogen initially and after 48 hours, with a quick utilization of polyphosphate. During the 168 hours of anaerobic endogenous respiration, glycogen decreased from 99.1 mg C/g VSS to 34.3 mg C/g VSS in SBR-H and the phosphorus released was 60.2 mg P/g VSS, while in SBR-L, glycogen decreased from 115.5 mg C/g VSS to 21.2 mg C/g VSS and the phosphorus released was 91.8 mg P/g VSS. During the 168 hours of aerobic endogenous respiration, glycogen decreased from 99.6 mg C/g VSS to 23.7 mg C/g VSS in SBR-H and the phosphorus released was 52.7 mg P/g VSS, while in SBR-L, glycogen decreased from 115.0 mg C/g VSS to 38.7 mg C/g VSS in SBR-H and the phosphorus released was 89.3 mg P/g VSS. These results showed that, during endogenous respiration, PAOs acclimated under the low influent organic carbon conditions utilized a higher amount of polymers than those of PAOs acclimated under a high influent acetate concentration.

During the anaerobic endogenous respiration, when the glycogen concentration decreased to 67.1 mg C/g VSS in SBR-H and 85.4 mg C/g VSS in SBR-L, degradation of polyphosphate occurred rapidly while this process was inhibited therebefore. During the aerobic endogenous respiration, when the glycogen concentration decreased to 81.8 mg C/g VSS in SBR-H and 87.2 mg C/g VSS in SBR-L, degradation of polyphosphate also occurred significantly with a high increase in the liquid phosphate concentration. These results showed that degradation of polyphosphate during endogenous respiration was controlled by the glycogen concentration and glycogen was firstly utilized as the energy source. When the glycogen concentration reached around 80 ± 10 mg C/g VSS, degradation of polyphosphate occurred. These showed that PAOs might prefer to utilize glycogen during the endogenous respiration for maintenance purposes [8, 20, 22, 23] and then utilize polyphosphate when the glycogen decreased to a certain level. Lopez et al. [8] also found that during the aerobic endogenous respiration, PAOs utilized PHA, glycogen, and polyphosphate sequentially, and phosphate release occurred after 16 hours of aerobic endogenous respiration.

For dynamics of all parameters during endogenous respiration, they were regressed by the first-order degradation equation and the results are shown in Table 2. There was no significant difference for biomass decay under anaerobic and aerobic conditions, which were close to those of 0.015–0.032 1/d obtained by Lu et al. [23], while they were lower than those of 0.14-0.15 1/d obtained in other studies [8, 20]. It should be noticed that, in most studies, biomass during decay was represented by the decrease in the concentration of VSS, while different environmental conditions might induce different intracellular or extracellular polymer concentrations, which might affect the description of biomass decay by using the parameter of VSS. Compared with the decay of biomass, decay coefficients of intracellular polymers were much higher. In addition, the degradation coefficient of glycogen was higher than that of polyphosphate. These results showed that during endogenous respiration, microorganisms would prefer to utilize stored polymers to maintain their activities. Comparing between degradation coefficients during both anaerobic and aerobic endogenous respirations, it was surprising to see there was not much difference for both decay of biomass and polymers.

During the anaerobic endogenous respiration, the degradation of intracellular carbohydrate in SBR-L was higher than that in SBR-H, which could be due to a slightly earlier utilization of polyphosphate in SBR-H and this might retard the utilization of glycogen, while under the aerobic endogenous respiration, the degradation of intracellular carbohydrate was slightly lower in SBR-L than that in SBR-H, which could be due to the fact that PAOs in SBR-L experienced starve conditions longer than those in SBR-H during acclimation, and they might had been used to the starve conditions better. For carbohydrate, there was a significant difference in biokinetics by using the total carbohydrate or the intracellular carbohydrate, confirming results from the previous study of Wu and Rodgers [20]. The anaerobic intracellular carbohydrate degradation rate was lower than that obtained by Wu and Rodgers [20], while similar results were obtained during the aerobic endogenous respiration. In the present study, PAOs were acclimated and all experiments were carried out at 25°C, while PAOs are psychrophile [24, 25]. The high temperature used in the present study might cause the decreased biokinetic activities, including a low anaerobic intracellular degradation coefficient.

## 4. Conclusions

Dynamics of polymers of PAOs under different organic carbon concentrations, including typical cycles, anaerobic metabolism with supply of excess organic carbon, and both anaerobic and aerobic endogenous respiration, were examined. Influent organic carbon concentrations had little influence on the yield of PAOs and the anaerobic polyphosphate release, while a low influent organic carbon concentration favoured the acclimation of PAOs and induced a high production of extracellular carbohydrate. During both anaerobic and aerobic endogenous respirations, when glycogen decreased to below around 80 ± 10 mg C/g VSS, PAOs began to utilize polyphosphate significantly. Regressed by the first-order reaction model, the degradation of glycogen possessed the highest reaction rate and was then followed by polyphosphate, while biomass decay had the lowest reaction rate.

## Figures and Tables

**Table 1:** Regressed biokinetic coefficients of polymers of PAOs under anaerobic conditions with the supply of excess organic carbon.

	Phase A: 0–50 min		Phase B: 50–110 min	
	SBR-H	SBR-L	SBR-H	SBR-L
*r*_PHB-C/NaAc-C_ (mg/mg)	0.97 (0.98)	1.08 (0.99)	0.72 (0.97)	0.66 (0.87)
*r*_PO4-P/NaAc-C_ (mg/mg)	1.03 (0.98)	1.44 (0.97)	1.05 (0.95)	1.66 (0.95)
*r*_Glycogen-C/NaAc-C_ (mg/mg)	0.85 (0.90)	1.10 (0.92)	0.35 (0.90)	0.28 (0.94)

**Table 2:** Regressed biokinetics of biomass or polymers by the first-order equation under both anaerobic and aerobic endogenous respirations (1/d).

	SBR-H		SBR-L	
	Anaerobic	Aerobic	Anaerobic	Aerobic
SS	0.074 (0.98)	0.070 (0.98)	0.074 (0.94)	0.082 (0.95)
VSS	0.041 (0.86)	0.048 (0.89)	0.038 (0.82)	0.058 (0.90)
Total carbohydrate	0.034 (0.65)	0.074 (0.92)	0.029 (0.56)	0.034 (0.67)
Internal carbohydrate	0.137 (0.96)	0.226 (0.96)	0.209 (0.93)	0.194 (0.98)
Polyphosphate	0.098 (0.94)	0.084 (0.97)	0.089 (0.99)	0.091 (0.99)
